# A Year in Review: Atrial Fibrillation 2022

**DOI:** 10.19102/icrm.2023.14017

**Published:** 2023-01-15

**Authors:** Mayank Sardana, Ankur A. Karnik, Rahul N. Doshi

**Affiliations:** ^1^Complex Arrhythmia Management, Cardiovascular Center of Excellence, HonorHealth Cardiac Arrhythmia Group, HonorHealth Medical Group, HonorHealth, Scottsdale, AZ, USA; ^2^University of Arizona College of Medicine Phoenix, Phoenix, AZ, USA

**Keywords:** 2022, atrial fibrillation, commentary, review

## Introduction

Atrial fibrillation (AF) continues to dominate the tumultuous electrophysiology world. While more patients than ever undergo life-saving treatments, reimbursement cuts threaten our field adversely. As always, our community will rally behind the exciting new developments in treatment strategies and let the science drive our field forward. We should be especially proud that the pandemic did not halt science in electrophysiology, as evident by the important results we will review.

## Catheter ablation for atrial fibrillation

The year 2022 did not disappoint—several critical studies were presented and published for AF ablation. We describe some notable ones below.

### Empirical box isolation of posterior wall: is there a role in persistent atrial fibrillation?

The Catheter Ablation for Persistent Atrial Fibrillation (CAPLA) multicenter randomized trial of pulmonary vein isolation (PVI) versus PVI with posterior wall isolation (PWI) was presented at the European Society of Cardiology meeting (peer-reviewed publication still awaited). This trial randomized participants with persistent AF undergoing index AF ablation in a 1:1 manner to PVI versus PVI + left atrial PWI. There were 169 participants in each arm, and the sample was calculated assuming a benefit of 15% from adding PWI. PWI was performed using a box isolation approach, which has been previously criticized due to its high failure rate.^1^ The primary endpoint was freedom from atrial arrhythmias off anti-arrhythmic drugs at 12 months. Investigators observed no difference in freedom from atrial arrhythmias with PVI (52.4%) versus PVI + PWI (53.9%) with a hazard ratio (HR) of 1.01 (95% confidence interval [CI], 0.74–1.38; *P* = .96).

The findings from this trial were heavily discussed during the meeting and on various social media platforms. Proponents of PWI proposed that endocardial box isolation is an ineffective strategy with reconnection rates of up to 63%. Therefore, if nearly 3 in 5 participants receiving PVI + PWI did not achieve durable PWI, how can we say that PWI does not add to PVI in persistent AF? Others argued that this is now the third clinical trial that has shown the ineffectiveness of PWI in persistent AF.^2,3^ These findings, when combined with the lack of effectiveness noted in a trial of participants undergoing repeat ablation,^4^ make endocardial box isolation of the posterior wall a questionable strategy.

However, the truth is probably somewhere in between! Perhaps, we are not addressing the critical role of epicardial tissue by endocardial box isolation. The Convergence of Epicardial and Endocardial RF Ablation for the Treatment of Symptomatic Persistent AF (CONVERGE) trial showed that the addition of epicardial ablation via a subxiphoid/transdiaphragmatic approach significantly improves arrhythmia-free survival in patients with persistent and long-standing persistent AF when compared to endocardial ablation alone (PVI + roof line + cavotricuspid isthmus ablation).^5^ Whether an endocardial debulking approach or the use of alternate sources of energy, such as in cryoballoon^6^ or pulsed-field ablation (PFA), can provide more durable/transmural PWI and perhaps better clinical outcomes remains to be established in randomized trials.

### Ablation targeting fibrosis

Clearly, we have not been able to reach a consensus regarding the optimal ablation strategy for patients with persistent AF. However, can an individualized strategy targeting fibrotic substrate yield better results?

To that end, the Efficacy of Delayed Enhancement Magnetic Resonance Imaging-guided Ablation Versus Conventional Catheter Ablation of Atrial Fibrillation (DECAAF-2) trial specifically attempted to answer this question.^7^ A total of 843 participants with persistent AF were randomized from 44 centers around the world to PVI plus imaging-guided fibrosis ablation (intervention group) or PVI alone (control group). Atrial fibrosis was measured using the Utah protocol on cardiac magnetic resonance imaging (MRI) scans and, in the intervention group, the operators were instructed to either cover or encircle the areas of fibrosis. The intention-to-treat analyses did not reveal a significant difference in freedom from atrial arrhythmia at 12 months (57% in the intervention group vs. 54% in the control group). In subgroup analyses, participants with less fibrosis (grade I or II, <20% fibrosis) seemed to derive an advantage from targeting the areas of fibrosis with regard to arrhythmia recurrence. On the contrary, in those with advanced degrees of fibrosis undergoing imaging-guided ablation, there was a signal toward an increased risk of periprocedural stroke.

While the DECAAF-2 trial did not find any significant advantage of targeting areas of fibrosis, one of the criticisms is that the detection of atrial fibrosis on cardiac MRI is not reproducible,^8^ and, therefore, an MRI-guided approach to target fibrosis might not be the best strategy. The Low-voltage Myocardium-guided Ablation Trial of Persistent Atrial Fibrillation (ERASE-AF) trial studied the role of targeting the regions of low-voltage areas (LVAs) (as a surrogate for fibrosis) identified on electroanatomic maps (EAMs) during the ablation.^9^ In this multicenter randomized trial, 324 participants with persistent AF were randomized in a 1:1 manner to PVI alone (control group) versus PVI + LVA ablation (intervention group). Operators were instructed on specific ablation lines to be performed depending on the location of LVAs.

In contrast to the findings of the DECAAF-2 trial, this trial showed the superiority of PVI + LVA ablation compared to PVI alone (arrhythmia-free survival at 12 months, 65% vs. 50%; *P* = .006). All EAMs were generated in sinus rhythm, ie, the patients were cardioverted to sinus rhythm if they presented in AF. Of note, the participants were randomized before the baseline EAM was created, and only 1 of 3 participants assigned to the intervention arm had LVAs and received additional ablation, while the rest underwent PVI only. Surprisingly, better outcomes were also observed in participants in the intervention group who did not have LVAs (and therefore only received PVI) compared to those in the control group who did not have LVAs. This subgroup analysis raised the question of a systematic error. Another criticism was that there was no “waiting period” for the control group to detect acute reconnection of pulmonary veins. It is plausible that the intervention group had better outcomes as any acute pulmonary vein reconnections were recognized and addressed during the LVA ablation. Therefore, the better outcomes in the intervention group might just be because of more durable PVI. The Substrate Ablation in the Left Atrium During Sinus Rhythm II (STABLE-SR-II) trial leveraged a very similar LVA ablation strategy in patients with early-stage (most within 6 months) persistent AF.^10^ Contrary to ERASE-AF, the STABLE-SR-II trial showed no benefit of LVA ablation over PVI.

Taken together, these trials suggest that an individualized strategy for persistent AF ablation might yield better outcomes than the “one size fits all” strategy; however, the optimal way to ablate LVAs remains unknown. There is increasing interest in targeting areas of isochronal crowding, ie, deceleration zones, as an add-on strategy to PVI.^11^ The deceleration zones often co-localize to LVAs identified by voltage mapping. A randomized trial investigating the role of add-on LVA versus deceleration zone ablation would certainly be welcome!

### Early pulmonary vein isolation is the goal with paroxysmal atrial fibrillation

Several trials over the last 2 years have focused on the role of early rhythm control (Early Treatment of Atrial Fibrillation for Stroke Prevention Trial 4 [EAST-AFNET 4]) and early ablation (STOP-AF, Early Aggressive Invasive Intervention for Atrial Fibrillation [EARLY-AF], CRYO-FIRST) for patients with paroxysmal AF.^12^ The follow-up study of the EARLY-AF trial, PROGRESSIVE-AF, was published this year.^13^ Briefly, 303 participants with newly diagnosed paroxysmal AF were randomized to rhythm control using cryoballoon ablation versus anti-arrhythmic drug therapy. Over 36 months of follow-up using continuous monitoring (loop recorders), 3 patients (1.9%) in the ablation group had an episode of persistent AF versus 11 patients (7.4%) in the drug therapy group (HR, 0.25 [95% CI, 0.09–0.70]). Recurrent atrial arrhythmias were observed in 56% of participants in the ablation group and 77% of participants in the drug therapy group. Furthermore, rates of hospitalization and serious adverse effects observed over 3 years were significantly lower for the ablation group versus the drug therapy group. This trial highly supports the emerging notion that early AF ablation leads to a delay in the progression of AF.

## Non-ablation therapies

### New developments in stroke prevention

Direct oral anticoagulants (DOACs) have become the mainstay of stroke prevention in non-valvular AF, supplanting vitamin K antagonists (VKAs) as first-line therapy in both European and U.S. guidelines.^14,15^ However, a robust head-to-head comparison of available agents has been lacking until now. A multinational population-based cohort study of 527,226 individuals with newly diagnosed AF has helped to answer this question.^16^ Standardized electronic health databases covering 221 million people in France, Germany, the United Kingdom, and the United States identified patients who were newly diagnosed with AF and received a new prescription for a DOAC. Apixaban was associated with a lower risk of gastrointestinal bleeding than dabigatran, rivaroxaban, and edoxaban (HR, 0.81 [95% CI, 0.7–0.94]; HR, 0.72 [95% CI, 0.66–0.79]; and HR, 0.77 [95% CI, 0.66–0.91], respectively). All agents had similar rates of ischemic stroke or systemic embolism, intracranial hemorrhage, and all-cause mortality. These findings persisted in those >80 years of age and those with chronic kidney disease.

Trials that established the efficacy of both VKAs and DOACs for stroke prevention in AF excluded those with rheumatic heart disease.^17,18^ Given the limited data, current guidelines do not recommend DOACs for these patients.^14,15^ The Investigation of Rheumatic AF Treatment Using Vitamin K Antagonists, Rivaroxaban or Aspirin Studies (INVICTUS) trial has definitively put this issue to rest.^19^ A total of 4,565 patients with rheumatic heart disease, AF, and a CHA_2_DS_2_-VASc score of ≥2 points were randomly assigned to either dose-adjusted VKA therapy or rivaroxaban. Eighty-two percent had moderate-to-severe mitral stenosis. Compared to those on DOACs, those on VKA therapy had a lower cumulative incidence of stroke, systemic embolization, myocardial infarction, or death from a vascular or unknown cause (*P* < .001) and longer survival times (*P* < .0001) without an increased risk of bleeding. Notably, the VKA group had more physician engagement, which could have led to better overall care and fewer strokes and deaths.

Despite their safety profile, DOACs increase the bleeding risk, and real-world data reveal a higher rate of emergency admissions for bleeding since their introduction.^20^ Traditionally, hemostasis and thrombosis were felt to be inextricably linked such that interventions to reduce thrombosis induce bleeding. However, recent evidence demonstrates that factor XIa plays an important role in thrombosis but a minor role in hemostasis and would be an attractive candidate for stroke prevention. Indeed, patients with factor XIa deficiency do not have an elevated risk of intracranial or gastrointestinal bleeding.^21^ The Safety of the Oral Factor XIa Inhibitor Asundexian Compared with Apixaban in Patients with Atrial Fibrillation (PACIFIC-AF) trial is the first-in-human trial of asundexian, a factor XIa inhibitor.^22^ A total of 755 enrolled patients with a CHA_2_DS_2_-VASc score of ≥2 points in men or ≥3 points in women were randomly assigned to asundexian or apixaban, and it was determined that rates of bleeding were lower in the asundexian group (HR, 0.33 [95% CI, 0.09–0.97]). The study was not powered to test differences in rates of thrombosis between groups but nonetheless represents exciting progress.

Up to 32% of patients undergoing left atrial appendage closure (LAAC) with the WATCHMAN device (Boston Scientific Corp., Marlborough, MA, USA) have incomplete closure.^23^ In the U.S. Food and Drug Administration clinical trials of LAAC, a peri-device leak (PDL) of ≤5 mm was considered acceptable at both implant and 45 days after implant. However, the long-term consequences of PDL on stroke risk have not been well studied. A cohort study of 1,054 patients using combined data from the Percutaneous Closure of the Left Atrial Appendage Versus Warfarin Therapy for Prevention of Stroke in Patients with Atrial Fibrillation (PROTECT-AF), Evaluation of the WATCHMAN Left Atrial Appendage (LAA) Closure Device in Patients with Atrial Fibrillation Versus Long Term Warfarin Therapy (PREVAIL), and Carbohydrate and Prostate Study 2 (CAPS2) trials sought to answer this question.^24^ In this study, the mean age was 74 ± 8.3 years and the CHA_2_DS_2_-VASc score was 4.1 ± 1.4 points. The presence of PDL ≤ 5 mm as assessed by transesophageal echocardiography (TEE) at 1 year (but not at 45 days) was associated with an increased 5-year risk of ischemic stroke or systemic embolism compared to the risk of those who had no leak (9.5% vs. 5.1%, *P* = .014). This study highlights the importance of optimizing device positioning at implant and considering closing PDLs, especially in those with PDLs who have suffered thromboembolic events. On a bright note, recent data show that the WATCHMAN FLX and Amulet (Abbott, Chicago, IL, USA) devices have reduced 1-year PDL rates.^25,26^

Also new among LAAC data, 4-year outcomes of the Left Atrial Appendage Closure vs. Novel Anticoagulation Agents in Atrial Fibrillation (PRAGUE-17) trial were released.^27^ This was a randomized, non-inferiority, prospective trial of 402 patients comparing percutaneous LAAC (WATCHMAN or Amulet) to DOACs (95% apixaban). The mean CHA_2_DS_2_-VASc score was 4.7 ± 1.5 points, and the mean HAS-BLED score was 3.1 ± 0.9 points. After 3.5 years, LAAC was non-inferior to DOACs for the primary endpoint (cerebrovascular event, systemic embolism, cardiovascular death, bleeding, or complications; *P* = .27) and led to fewer non-procedural bleeding events (*P* = .028). Notably, 9 patients (4.5%) in the LAAC group experienced significant procedural complications, including 2 procedure-associated deaths.^28^

Last year, the Amulet-IDE trial demonstrated that the Amulet occluder was non-inferior for safety and effectiveness of stroke prevention for non-valvular AF compared to the WATCHMAN device and superior for LAA occlusion.^26^ The Comparison of Amulet Versus Watchman/FLX Device in Patients Undergoing Left Atrial Appendage Closure (SWISS-APERO) trial was a multicenter, randomized, controlled trial of 221 patients comparing the Amulet and WATCHMAN FLX devices in terms of sealing capacity, procedural complications, and short-term clinical outcome.^29^ At 45 days, PDLs (as assessed by TEE) were larger with WATCHMAN FLX (27.5% vs. 13.7%, *P* = .020), although none were >5 mm. There were also fewer device-related thrombus events associated with Amulet use (2.1% vs. 5.5%) as assessed by TEE. However, major procedure-related complications occurred more frequently in the Amulet group (9.0% vs. 2.7%, *P* = .047), mostly because of bleeding, suggestive perhaps of site selection or structural program experience.

### Prevention of postoperative atrial fibrillation

Postoperative AF (POAF) occurs in 15%–42% of patients following cardiac surgery.^30^ Inflammation, cardiac ischemia, and sympathetic activation in the setting of abnormal atrial substrate likely contribute to POAF.^31^ Prophylactic pharmacotherapy can reduce the length of stay and reduce hospital costs by $1,250 USD.^32^ Meta-analyses have shown that amiodarone, atrial pacing, posterior pericardiotomy, landiolol, and colchicine significantly reduce POAF after cardiac surgery.^32–34^ In a randomized, double-blinded, placebo-controlled trial of 200 patients undergoing coronary artery bypass graft (CABG) surgery, berberine was found to reduce POAF at 7 days from 35% to 20% (*P* = .0143).^35^ Berberine is a quaternary ammonium salt and traditional Chinese medical herb that has anti-inflammatory and possibly class III anti-arrhythmic properties. In another randomized, double-blinded, placebo-controlled study of 200 patients undergoing CABG surgery, preoperative oral magnesium significantly reduced POAF (10% vs. 22% placebo; HR, 0.45 [95% CI, 0.23–0.91]).^36^ Multicenter trials are needed to confirm these interesting findings.

### Preventing the progression of paroxysmal atrial fibrillation

Calcium channel blockers have been shown to reduce tachycardia-mediated atrial remodeling in AF.^37^ In vagally mediated AF, verapamil has been found to reduce patient progression to persistent AF compared to β-blockers or digoxin.^38^ Integrated Chronic Care Program at Specialized AF Clinic Versus Usual Care in Patients with Atrial Fibrillation (RACE4) was a randomized trial that evaluated nurse-integrated versus standard care for patients with AF.^39^ In a post hoc analysis of 666 patients with paroxysmal AF, those on verapamil were significantly less likely than those on β-blockers or no rate control to require electric/chemical cardioversion or ablation (combined endpoint, *P* = .038).^40^ Atrial cardiomyopathy is an arrhythmic substrate for AF,^41^ and, as our understanding deepens, so too will our therapeutics improve.

## Conclusion

The year 2022 has provided a wealth of science to refine our treatment strategies for AF. We anxiously await what the new year has in store! More on PFA, alternative mapping and ablation strategies, and alternative therapies to come!
